# A SYSTEMS APPROACH ON THE IMPACT OF ENVIRONMENTAL RACISM AND CLIMATE CHANGE ON RESIDENTS OF VINE CITY IN ATLANTA GA

**DOI:** 10.1093/geroni/igad104.3727

**Published:** 2023-12-21

**Authors:** Kellie Mayfield, Kehinde Akintola, Sophia Foley, Angelique Willis, Miranda Cook

**Affiliations:** Georgia State University, Atlanta, Georgia, United States; Georgia State University, Atlanta, Georgia, United States; Georgia State University, Atlanta, Georgia, United States; Georgia State University, Atlanta, Georgia, United States; Open Hand Atlanta, Atlanta, Georgia, United States

## Abstract

There are three interlocking factors impacting the health of older-adults. First is food access, the ecologies of their community and the systems they interact with. In the community of this study the local grocery store closed limiting food access. The ecology of the environment is a heat island; which leads to the incidence of higher mortality amongst other health effects. The interacting systems are the economy, ageism, classism, racism, and, sexism. This conceptual model encapsulates the environment impacting one community of older adults in Atlanta, GA from a mixed methods study employing survey and photoelicitation with the purpose of examining the facilitators and barriers to healthy eating by senior-center clients served by Open Hand Atlanta; a meals and nutrition education provider. Descriptive statistics showed food insecurity to be double that of older adults statewide (19%), 7.9 % reporting consuming at least 5 cups of fruit and vegetables per day, and participants were mostly black women (79 %) . Additionally, residents also live in a community where a portion has been deemed a toxic Superfund site because of elevated levels of lead in soil. The model, therefore, contributes to a comprehensive understanding of how the health of older adults is impacted by a multitude of systems and climate change. It can be used as a guide for other communities, community-dwelling older adults, and older-adult service providers. Furthermore, this model illustrates the systems impacting the health of older southern adults.

